# Economic benefits and costs of surgery for filarial hydrocele in Malawi

**DOI:** 10.1371/journal.pntd.0008003

**Published:** 2020-03-25

**Authors:** Larry Sawers, Eileen Stillwaggon, John Chiphwanya, Square Z. Mkwanda, Hannah Betts, Sarah Martindale, Louise A. Kelly-Hope

**Affiliations:** 1 Department of Economics, American University, Washington, District of Columbia, United States of America; 2 Department of Economics, Gettysburg College, Gettysburg, Pennsylvania, United States of America; 3 Ministry of Health, Lilongwe, Malawi; 4 Centre for Neglected Tropical Diseases, Department of Tropical Disease Biology, Liverpool School of Tropical Medicine, Liverpool, United Kingdom; Hitit University, Faculty of Medicine, TURKEY

## Abstract

**Background:**

Lymphatic filariasis (LF) is endemic in 72 countries of Africa, Asia, Oceania, and the Americas. An estimated 25 million men live with the disabling effects of filarial hydrocele. Hydrocele can be corrected with surgery with few complications. For most men, hydrocelectomy reduces or corrects filarial hydrocele and permits them to resume regular activities of daily living and gainful employment.

**Methodology and principal findings:**

This study measures the economic loss due to filarial hydrocele and the benefits of hydrocelectomy and is based on pre- and post-operative surveys of patients in southern Malawi. We find the average number of days of work lost due to filarial hydrocele and daily earnings for men in rural Malawi. We calculate average annual lost earnings and find the present discounted value for all years from the time of surgery to the end of working life. We estimate the total costs of surgery. We compare the benefit of the work capacity restored to the costs of surgery to determine the benefit-cost ratio. For men younger than 65 years old, the average annual earnings loss attributed to hydrocele is US$126. The average discounted present value of lifetime earnings loss for those men is US$1684. The average budgetary cost of the hydrocelectomy is US$68. The ratio of the benefit of surgery to its costs is US$1684/US$68 or 24.8. Sensitivity analysis demonstrates that the results are robust to variations in cost of surgery and length of working life.

**Conclusion:**

The lifetime benefits of hydrocelectomy–to the man, his family, and his community–far exceed the costs of repairing the hydrocele. Scaling up subsidies to hydrocelectomy campaigns should be a priority for governments and international aid organizations to prevent and alleviate disability and lost earnings that aggravate poverty among the many millions of men with filarial hydrocele.

## Author summary

Lymphatic filariasis (LF) is endemic in 72 countries of Africa, Asia, Oceania, and the Americas. It causes painful, disabling hydrocele in an estimated 25 million men. Hydrocele leads to reduced mobility, social exclusion, and depression, all of which limit the men’s ability to work. Filarial hydrocele can be corrected with surgery with few complications. We measured the economic loss due to hydrocele and the benefits of hydrocelectomy in two districts in southern Malawi. We calculated lost earnings over the lifetime due to filarial hydrocele and compared it to the costs of surgery to determine the benefit-cost ratio. The ratio of the benefit of surgery to its cost is US$1684/US$68 or 24.8. The results are robust to variations in cost of surgery and length of working life. Scaling up subsidies to hydrocelectomy campaigns should be a priority for governments and international aid organizations to prevent and alleviate disability and lost earnings that aggravate poverty among the many millions of men with filarial hydrocele, their families, and their communities.

## Introduction

### Lymphatic filariasis and its sequelae

Lymphatic filariasis (LF) is endemic in 72 countries of Africa, Asia, Oceania, and the Americas.[1] Various species of mosquitoes, depending on world region, transmit the parasites, *Wuchereria bancrofti*, *Brugia malayi*, and *Brugia timori*, to humans. As of 2017, the Global Programme to Eliminate Lymphatic Filariasis (GPELF) had been successful in promoting mass drug administration (MDA) in 67 countries with the goal of stopping new infections–the first “pillar” of the GPELF program–and 21 countries had already completed MDA.[1] The second pillar of the GPELF addresses the needs of people already infected. As many as 40 million people live with the disabling effects of LF, including about 15 million persons with chronic lymphedema, primarily of the legs, but also of the arms, breasts, and scrotum, and about 25 million men with hydrocele.[2] In addition, tens of millions of persons infected with LF are still at risk of developing lymphedema or hydrocele due to damage to the lymphatic system caused by the parasite.[3]

By 2017, only 38 LF-endemic countries had reported services to manage morbidity and prevent disability among affected persons.[1] The vast majority of those programs address only lymphedema.[4] Simple habits of leg hygiene and infection control can slow or stop the progression of lymphedema by reducing the frequency of episodes of infection, called acute dermatolymphangioadenitis (ADLA). Both lymphedema and ADLA diminish productivity.[4–12] Community programs to promote leg hygiene have been successful at trivial cost.[13–15] An economic study of such a community program in Odisha, India estimated that the lifetime benefits of disability prevented, restored work capacity, and reduced medical expense would be 130 times the cost of the intervention.[16]

In regions endemic with *W*. *bancrofti*, the most common manifestation of LF is hydrocele.[17] Hydrocele is the accumulation of fluid around the testis in the tunica vaginalis, expanding the fluid volume within the scrotal sac due to blockage or dysfunction of the lymphatic vessels.[4, 18] Hydrocele can be corrected with surgery with few complications. For most men hydrocelectomy reduces or corrects filarial hydrocele and permits resumption of regular activities of daily living and gainful employment.[19–25]

A few countries have organized intensive campaigns in which scores of men are recruited for surgery during a few days or weeks. The present study reports on one such campaign in 2015 in Malawi and evaluates the lifetime economic costs of filarial hydrocele and the lifetime benefits of the surgery from a societal perspective compared to the cost of the surgery. The calculations include only earnings losses and gains for the patients and costs of surgery, omitting costs or gains to family caregivers and community members, which were not measured.

### The effects of filarial hydrocele and of hydrocelectomy

Hydrocele is frequently a painful condition that leads to reduced mobility, social exclusion, and depression, all of which reduce the ability to engage in productive activities.[4, 19–25] The productivity loss takes different forms. Some men with hydrocele are unable to work; others may work fewer days per week or hours per day and may be less productive while working. The principal obstacles to undergoing hydrocelectomy reported by men in resource-poor countries include its substantial cost and lack of nearby surgical facilities.[26] Filarial hydrocele disproportionately affects poor men, for whom the cost of hydrocelectomy can be prohibitive. Men in rural areas have less access to surgical facilities and less correct information about the effectiveness of surgery.

The economic impact of filarial hydrocele has been investigated in several countries. A study in east central India (Odisha State) in 2002 found that men with filarial hydrocele worked 14% fewer hours per day than controls.[25] Another study in 1999 in south India found filarial hydrocele patients worked 18% fewer hours per day than controls.[27] A third study in 1996 examined the productivity of 39 male weavers in south India, of whom 29 had hydrocele and 12 had lymphedema. The research compared them with matched controls without chronic lymphatic filariasis. The men worked similar numbers of hours, but those with hydrocele or lymphedema produced 27% less cloth.[28] There are also qualitative studies of productivity losses from filarial hydrocele. A 1996 study in Ghana, for example, reported that people with chronic manifestations of lymphatic filariasis (lymphedema and hydrocele) worked only in short bursts or turned to sedentary but less remunerative jobs, such as basket weaving.[22] See also [19–21, 23, 29].

Efficacy, complications, and serious adverse events are also important considerations in evaluating the benefits and costs of these surgical campaigns, as with any medical or public health intervention. Stanton *et al*.[30] interviewed 40 men in southern Malawi who had undergone hydrocelectomy 6 months to 2 years earlier. They found that very few men reported any problem with pain, mobility, anxiety, engagement in usual activities, or social participation after surgery. Responding to questions about each of those 5 issues, only a single respondent reported more than a mild problem. Ahorlu *et al*. reported on their 1999 study of 40 men in a hydrocelectomy campaign in Ghana and found that the men reported “no complications during and after the surgery [and] everybody recovered well.”[31] In contrast, Thomas *et al*. reported on 5 “mass surgery weeks” between 2002 and 2005 in Nigeria that performed 301 hydrocelectomies; follow-up of 115 of those patients in 2005 found that 7% had a recurrence of the previous condition and 4% developed a new hydrocele.[32] In 2011, Mante and Gueye reported on 3,000 hydrocelectomies in 10 West African countries among which the recurrence rate varied between 3% and 5%.[33]

A study of hydrocelectomy in Edmonton, Canada with a substantial (9.3%) treatment failure rate[34] has been cited in research on the costs of filarial hydrocelectomy.[24] None of the hydroceles in Edmonton, however, was of filarial origin; they were caused by trauma, infection, or tumor. The procedure was performed in an outpatient setting, with an annual average of 3.5 hydrocelectomies per surgeon. The high failure rate in the Edmonton study thus sheds little light on campaigns carried out on an inpatient basis in a Level II hospital with surgeons specifically trained to treat filarial hydrocele, as was recommended in a 2002 meeting of the GPELF.[35]

### LF prevention and treatment in Malawi

Malawi, one of the world’s poorest countries, launched a preventive chemotherapy campaign in 2008 and has now completed at least five rounds of MDA. The country conducted the Transmission Assessment Survey (TAS) and carries out surveillance in every endemic district.[1] In 2015 a surgery campaign and surveys were coordinated by the Malawi Ministry of Health, with technical and financial support from the Centre for Neglected Tropical Diseases (CNTD) at the Liverpool School of Tropical Medicine (LSTM). (For a detailed description of the campaign’s methods, see [36].)

## Methods

This study measures the economic loss due to filarial hydrocele and the benefits of hydrocelectomy and is based on pre- and post-operative surveys of patients during a campaign in late 2015 in Malawi.

### Ethics statement

Ethical approval for the research was granted by the Institutional Review Board of the Liverpool School of Tropical Medicine, Liverpool, UK (15.047) and the National Health Sciences Research Committee, Ministry of Health, Malawi (15/3/1406). The data used for this economic analysis were stripped of personal identifiers and thus the economic analysis of the data did not require additional ethical clearance.

### The survey

During the hydrocelectomy campaign in Malawi, a pre-operative survey was administered to 201 men in December 2015. The interviews were conducted in 6 hospitals in Chikwawa and Nsanje Districts, which have the highest poverty rates in the country.[37] While nearly 85% of Malawi’s population lives in rural areas, more than 97% of Chikwawa’s population and 92% of Nsanje’s population live in rural areas.[38] These districts are the most severely affected by lymphatic filariasis in Malawi.[39, 40] Follow-up interviews were conducted 3 months and 6 months after the surgery; 152 men (76%) were selected randomly, stratified across the six hospitals. Of those 152 respondents, 137 (68%) could be located for the 6-month follow-up.[36]

The questionnaire was developed by the LSTM research team at the CNTD and translated into the local language by the Malawi LF Programme team. Informed consent was obtained with a written form that was signed and dated by the patient. Tablets for recording responses were given to nurses trained for this task who interviewed patients during pre-surgical assessment. Hydrocelectomy patients were examined by a nurse who evaluated the size of the hydrocele, following the classification of Capuano and Capuano.[41] The men were assigned to one of 6 stages according to the size of the scrotum. The data were collated electronically via Open Data Kit (ODK, https://opendatakit.org/) software and downloaded into Excel spread sheets (Microsoft Corporation, Redmond, WA, USA).

### Measuring productivity loss from filarial hydrocele in Malawi

Our measure of productivity loss from untreated filarial hydrocele was based on the following question in the pre-operative survey: “How many days (add partial days together to make full days) [have you] been unable to work in the last month due to your hydrocele?” In Malawi, the number of work days in a month is conventionally considered to be 26.[42, 43] For the 11% of respondents who reported more than 26 work days lost to hydrocele disability in the previous month, we record their days lost as 26.

We used other questions in the survey to test the validity of the days-of-work-lost question as our measure of productivity loss. We calculated summary disability scores for mobility, pain, and participation in usual activities, both taken separately and summed into a single measure. (Each of the 3 disability scores was based on 4, 5, or 6 questions for a total of 45 questions. Responses were coded as follows: no problem = 0, mild problem = 1, moderate problem = 2, and severe problem = 3. For example, a respondent reporting “moderate problem” on all 45 questions would have a summary score of 90. (See [36] for more information about disability scores reported by respondents.)

In a simple linear regression with days of work lost as the dependent variable and the sum of the 3 disability measures as the independent variable, the *t*-statistic was 6.1 (with an adjusted R-squared of 0.16, significant at the .0000 level). That correlation suggests that the days-of-work-lost variable is a useful measure of the work-capacity or productivity dimension of the economic burden of filarial hydrocele. In contrast, hydrocele stage did not have a statistically significant relationship with either the sum of the 3 disability measures (adjusted R-squared of 0.01, significant at the 0.08 level) or with days of work lost (adjusted R-squared of 0.00 significant at the 0.33 level). All regressions in this work were estimated using STATA/SE 15.1 (StataCorp., College Station TX).

### Calculating daily earnings loss from filarial hydrocele

Because there are no studies that specifically measure rural male earnings in Malawi, we combine 2 methods to estimate the average value of a day’s earnings in rural Malawi, which allows us to assign a monetary value to each respondent’s daily productivity loss due to filarial hydrocele. In rural parts of a country such as Malawi, income typically comes to the family in a variety of ways that do not lend themselves to quantitative measurement. Measuring the loss in earning capacity imposed by filarial hydrocele is especially challenging in agricultural areas where an important share of household income consists of food grown and consumed by the family. Another form of income is surplus agricultural production sold for cash or bartered. In many families, only a small share of income takes the form of cash wages generated by either occasional or regular employment. What follows presents 2 ways of estimating average daily earnings of hydrocele patients.

The hydrocelectomy campaign in Malawi took place at the end of 2015. The last follow-up interviews were conducted in June 2016. We begin our calculations of the stream of lifetime earnings losses in July 2016.

#### Daily earnings estimate 1

Our first measure is the average income of people in Malawi who live in poverty, as determined by the World Bank. The World Bank’s global measure of poverty established the poverty line in 2011 at US$1.90 a day (PPP).[44] (PPP or purchasing power parity adjusts for different patterns of prices among countries.) In Malawi in 2011, 71% of the population lived on less than US$1.90 PPP a day. The World Bank calculated the average daily income of people living below the poverty line in Malawi in 2011 to be US$1.26. Adjusting for dollar inflation between 2011 and 2016, we find the average daily income of those living below the poverty line in Malawi was US$1.34 in mid-2016.[45]

#### Daily earnings estimate 2

The second way we measure average daily earnings uses data from the *Malawi Labour Force Survey 2013*.[46, 47] The 2013 survey (in both the Main Report and the Key Findings Report) states that “monthly gross income” in rural areas (also described in the report as “monthly wages” or “monthly earnings”) was 12,000 kwacha. At the average exchange rate at the time of that survey (December 2012 to March 2013), 12,000 kwacha was equivalent to US$34.29. Dividing by the conventional 26 work days per month in Malawi[42] yields an average rural daily earnings of US$1.32 in 2013. Adjusting for dollar inflation between 2013 and 2016, we find the average rural daily earnings were US$1.38 in 2016.[45, 48]

These 2 measures of the value of a day’s earnings in rural Malawi (US$1.34 and US$1.38) average US$1.36. Both measures underestimate men’s earnings because no distinction is made between men’s and women’s earnings. In Malawi, men’s median income is reported to be 52% higher than women’s.[46] Assuming men comprise half the work force (paid and unpaid), our estimate of men’s average daily earnings in rural Malawi is US$1.64.

### Calculating lifetime earnings loss from filarial hydrocele

We determine the value of the annual earnings loss due to filarial hydrocele for men in the survey by multiplying each respondent’s reported work days lost in the previous month by US$1.64 and multiplying that monthly figure by 12. To calculate lifetime earnings loss due to filarial hydrocele, we estimate the number of years that respondents can be expected to remain in the workforce after hydrocelectomy.

#### Age progression

If hydrocele stage progressed with age, it could be desirable to adjust the men’s current disability for expected future disability. In the survey, however, the correlation between age and stage of hydrocele (1 through 6) as assessed by medical staff was not statistically significant. In a simple linear bivariate regression of age and stage for men under 65 (the age for which we calculate earnings lost or gained), the R-squared was .001. Furthermore, Table 1 suggests no age progression of hydrocele severity except for the 10 men in the survey younger than 30. Accordingly, we assumed no age progression of hydrocele in our calculations of future loss of productivity from hydrocele and gains in productivity from surgery.

**Table 1:** Mean stage of hydrocele and patient age.

Age	Number of men	Mean stage
**20–29**	10	1.90
**30–39**	34	2.32
**40–49**	35	2.54
**50–59**	36	2.22
**60–65**	25	2.28

#### Aging out of the workforce

In Malawi in 2016, male life expectancy at birth was 61 years.[49] Since Chikwawa and Nsanje are the poorest regions of Malawi, life expectancy in those districts might be lower than the national average.[37] On the other hand, 69% of Malawian males who survive childhood live beyond the age of 60.[49] In addition, among the men in the survey who were 65 and older, only 3 reported no gainful employment. For a conservative estimate of future lost productivity, we assume that men in the survey will age out of the workforce at age 65 (or that on average men’s productivity tapers off over a period centered on 65 years). Subtracting each man’s age from 65 gives us his expected years of working life. We use each man’s annual earnings loss and his expected years of working life to find his expected lifetime earnings loss due to uncorrected filarial hydrocele.

#### Discounting

Assuming that an amount of money in the present is worth more than the same amount in the future, economists discount future costs and/or benefits. We apply the standard annual discount rate of 3% used in health economics to benefits and costs occurring beyond the current year.

#### Inflation and real earnings growth

To measure the value of men’s productivity losses until they age out of the workforce, we use the value of the dollar in July 2016, which was roughly six months after the surgery, when the men were fully recovered. Any subsequent nominal increase in men’s earnings due to inflation would not reflect the real value of the goods and services that earnings can actually buy. Furthermore, we do not project any future real earnings increases in Malawi since income growth there in the last two decades has been erratic. A fertilizer subsidy program in the early 2000s initially had substantial success in raising rural incomes and lifting the country out of famine, but income growth has since faltered.[50]

#### Lifetime earnings loss and the effect of hydrocelectomy

Our assumption that real annual earnings gains from hydrocelectomy are constant over the men’s working lives is supported by our finding of no reported treatment failures or recurrence of symptoms in the 6-month follow-up survey. In the pre-operative survey, the men were asked about disability imposed by their filarial hydrocele, and 84% reported mobility impairment, 81% reported difficulty in engaging in usual activities, 88% reported pain in their groin, 97% reported psychological problems, and 75% reported social problems due to hydrocele. The respondents in the post-operative survey reported that surgery almost completely eliminated the disability imposed by hydrocele. At the 6-month follow-up (*n* = 137), no respondents reported any mobility restriction, problems with usual activity, pain, social problems, or psychological issues. Given the success of hydrocelectomy reported by respondents in the survey, we assume that the surgery eliminated the earnings loss due to the inability to work once the recovery period (averaging 35 days) had elapsed.

The sum of discounted real earnings loss for every year until the end of a man’s working life gives the real present value of his lifetime earnings loss due to filarial hydrocele, which is equivalent to the projected earnings gained from a successful hydrocelectomy that restores the man’s productivity. We find the average for all men including those who reported no days of work lost due to hydrocele in the pre-operative survey.

We calculate the lifetime benefits of hydrocelectomy beginning in July 2016, by which time the six-month follow-up survey was completed, participants in the hydrocelectomy campaign had recovered from their surgeries, and respondents could report on the success or failure of the procedure. The lifetime benefits of the surgery are the earnings gained by eliminating the disability imposed by hydrocele.

### Costs of hydrocelectomy

The economic costs of hydrocelectomy include the costs of the surgery itself and the loss of earnings during recuperation. Both of those are one-time costs incurred in late 2015 and the first half of 2016 and are not discounted. The direct cost of the surgery, or budgetary cost, is the cost from the perspective of government or donor agencies funding the surgery. Economists, however, would add a second cost, the loss of earnings that the men bear during recuperation from the surgery.

#### Surgery costs

The per-patient cost of the surgery was UK£45 or US$68, as per the agreement between CNTD and the Malawi government.[48] This budgetary cost includes CNTD contributions to patient costs (accommodation, meals, travel, and registration fee) and medical supplies. The cost of the surgery excludes staff training (an investment in human capital, not a current expense) and monitoring costs (the cost of the follow-up surveys).

#### Earnings loss during recuperation

For patients who missed days of work while recuperating from the surgery, their foregone earnings are a cost of the surgery measured in societal, rather than strictly budgetary, terms. That lost work time, however, is a cost in an economic sense (opportunity cost) only to men who missed fewer days to hydrocele in the year before the surgery than they did during recovery in the year after the surgery. We find the average number of days in recovery for all men and calculate the opportunity cost for men who would have missed less work time had they not had the hydrocelectomy. Each work day lost was valued at US$1.64.

#### Benefit-cost ratios

We compare the economic benefit of work capacity restored through surgery (earnings gain) to the costs of surgery, both budgetary and economic, to calculate benefit-cost ratios.

#### Sensitivity analysis

To test the robustness of our results, we examine different assumptions about the length of men’s working lives and different estimates of the costs of hydrocelectomy. We calculate the real value of lifetime earnings gain from hydrocelectomy assuming that men leave the workforce at 60 and at 70 years. We use estimates by WHO of a range of hydrocelectomy costs from US$80 to US$360. (See [51] page 137. WHO does not report the source of the estimates or explain what costs are included. It is likely that these are the direct costs of surgery, or budgetary costs, and do not include lost earnings during recuperation.) We then calculate the benefit-cost ratios with those different assumptions about length of working life and cost of hydrocelectomy.

## Results

### Earnings loss

The average number of work days lost among the men younger than 65 was 6.4 per month or 76.8 per year. The value of each day of earnings lost was estimated to be $1.64, and thus the average annual earnings loss was US$126. That includes 67 men who reported no lost work days due to hydrocele. Assuming no future adverse events associated with the hydrocelectomy and no recurrence of hydrocele, the average discounted real present value of lifetime earnings loss for men younger than 65 years was US$1,684.

### Benefits of surgery

In the six-month follow-up survey, the 137 respondents were asked how many days (adding partial days together to make full days) they had been unable to work in the last month due to their groin. None of the respondents reported any days of work lost. The surgery eliminated the loss of earnings for all respondents due to the inability to work once the recovery period had elapsed.

### Costs of hydrocelectomy

Cost of foregone earnings during recovery was calculated from the pre- and post-operative surveys. In the 3-month post-operative survey (*n* = 152), average reported work time lost due to surgery was 35 days, with only 1 man reporting no work time lost. In the pre-operative survey, of the 140 men younger than 65 years, 49% had reported missing 36 or more days of work due to hydrocele in the year prior to surgery. For them, there was no net worktime loss due to inability to work during recuperation. The surgery created an opportunity cost only for the 51% of men who had reported missing fewer than 35 days of work due to hydrocele. The average earnings loss during recovery for the whole group of 140 men younger than 65 was US$28.

The total average cost, in economic terms, of the hydrocelectomy was the sum of US$68 (the medical or budgetary cost of the surgery) and US$28 (the average net loss of earnings during recovery), for a total cost of US$96.

### Benefit-cost ratio

A benefit-cost analysis compares the economic benefits of an intervention to the economic cost of the intervention. The average annual earnings loss due to hydrocele was US$126, nearly double the US$68 cost of the surgery. In other words, the surgery more than paid for itself in the first year after the procedure. But the benefits of the surgery continued for the rest of the men’s working lives. For the baseline assumption that the average age of departure from the workforce is 65 years, the ratio of the economic benefit of surgery to the direct or budgetary costs is US$1684/US$68 or 24.8 (Table 2). The ratio of the economic benefit of the surgery to the total economic cost is US$1684/US$96 or 17.5 (Table 2).

**Table 2:** Benefit-cost ratios at different ages of departure from the workforce and different costs of hydrocelectomy, budgetary costs and economic costs.

Hydrocelectomy Cost	Workforce departure at 60 years	Workforce departure at 65 years	Workforce departure at 70 years
**Actual costs Malawi campaign**			
**budgetary cost US$68**	23.2	24.8	28.5
**economic cost US$96**	16.4	17.5	20.2
**WHO cost estimates**			
**low estimate US$80**	19.7	21.1	24.2
**high estimate US$360**	4.4	4.7	5.4

### Sensitivity analysis

If, on average, men leave the workforce at age 60, the benefit-cost ratio ranges from 23.2 to 16.4 for budgetary and economic costs respectively, as shown in Table 2. If, on average, men leave the workforce at 70, the benefit-cost ratio ranges from 28.5 to 20.2 for budgetary and economic costs respectively.

Using WHO’s 2015 estimate of hydrocelectomy cost, we found the range of possible benefit-cost ratios for hydrocelectomy was 21.1 to 4.7 by varying cost from US$80 to US$360.[51] Simultaneously varying WHO’s cost of surgery and the average age of departure from the workforce from 60 years to 70 years, the benefit-cost ratio varied from 4.4 to 24.2, as shown in Table 2.

## Discussion

Hydrocele can limit one’s ability to engage in productive activities. That causes or aggravates economic distress and leads to depression and social exclusion, which can also limit productivity and undermine the wellbeing of the family and the larger community. Surgical correction of the condition reduces or eliminates filarial hydrocele’s negative effects after a few weeks or months of recovery. Compared to the lifetime economic costs of hydrocele, its surgical correction is not an especially costly intervention. Filarial hydrocele, however, is a condition that disproportionately affects poor men who find it difficult to pay for the procedure. In a study in Ghana, men with hydrocele reported that by far the most important barrier to hydrocelectomy was its cost.[20]

We found that, on average, men in the campaign were unable to work 6.4 days in the month prior to the interview due to their hydrocele. According to the men’s reports in the post-operative survey, the hydrocelectomy eliminated their disability with respect to mobility, pain, and social exclusion and, after an average 35-day recovery period, they would be able to work 6.4 more days per month until aging out of the workforce. The estimated real present value of those extra days of work to age 65 was US$1684 which was 24.8 times the direct cost of the hydrocelectomy. The results are robust to different assumptions about the length of the men’s working lives and the costs of hydrocelectomy.

Turner *et al*. is the only cost-effectiveness study of hydrocele. It projected that a typical hydrocelectomy would be classed as highly cost-effective if the cost of surgery was less than US$66 in 2014 dollars (US$67 in 2016).[24] Thus the surgery cost in the Malawi campaign, US$68, is virtually the same as the cost Turner *et al*. determined to be highly cost-effective. Turner *et al*. also determined that any surgery cost below US$398 (US$402 in 2016) would be classed as cost-effective, far above the Malawi cost and even exceeding WHO’s high estimate.

### Factors that could lead to overestimation of the benefit-cost ratio for hydrocelectomy

#### Surgical failure, complications, and serious adverse events

At the 6-month follow-up, no respondent reported any disability from hydrocele (judging by answers to 27 questions about the 6 domains of symptoms).[36] Accordingly, our calculations assumed no surgical failures. We describe above studies of other hydrocelectomy campaigns in Africa, some of which report surgical failure and adverse events[32, 33] and others that do not.[30, 31] Underestimation of treatment failure would exaggerate the economic benefits of hydrocelectomy, but we find no evidence of treatment failure in the Malawi campaign.

#### Weak labor demand

We assumed that all of the men could be reabsorbed into the workforce when they regained their ability to work. Weak labor demand, however, could limit the men’s ability to return to work in some labor markets, especially for men with specific skills. Nevertheless, in communities like Chikwawa and Nsanje, it appears that every able person works. Out of 201 respondents in our survey, only 5 men– 4 of whom were 60 or older–reported no earned income. Lack of labor demand does not appear to be an important barrier for men finding work in this largely agricultural region of Malawi.

### Factors that could lead to underestimation of the benefit-cost ratio for hydrocelectomy

#### Productivity and the wage

Wages can be a poor measure of the worker’s contribution to output because of the employer’s efforts to pay wages that are less than the value the worker produces. Moreover, if the employer takes that profit out of the community, it reduces spending in local shops or markets, diminishing the multiplier effects of the worker’s earnings on community income. Similarly, farmers who sell their crop to brokers may not receive the full value of their output, and the community would lose if those brokers were not local residents. Lacking a method of measuring workers’ output directly, earnings are the conventional, albeit conservative, proxy for productivity.

#### Intensity of work

We used days of work lost to measure the earnings loss due to hydrocele, but that likely understated substantially the productivity and earnings effects of hydrocele. The survey asked about days of work missed but did not ask about the intensity of work. Hydrocele can make it impossible for men to perform strenuous tasks that would be more productive and thus more remunerative. Our measure only included earnings loss due to absence from the workplace, not due to lower productivity or lower earnings per day.

#### Real wage stagnation

Our calculations assumed no increase in real earnings over the coming decades. This is a conservative assumption. If real wages do rise, then our estimate of real future earnings loss will underestimate actual earnings loss and the benefits of surgery.

#### Family coping and caregiving

A man with hydrocele may be less able to perform household tasks that are then taken over by family members, diminishing their productivity in other activities. Moreover, he may be so incapacitated that he requires help in activities of daily living, thereby reducing the amount of work that family members can do inside or outside the home or reducing the ability of child caregivers to attend school. He may have to pay non-family members for care and housework. Furthermore, reduced household income may make it difficult or impossible to pay for school tuition, uniforms, and other school supplies, reducing the earning capacity of the family years into the future. Since we have no way to measure them, these important second-order losses in economic well-being were not included in our analysis.

#### Societal effects

We did not include the multiplied effect of the men’s earnings loss on the community through reduced spending in local shops and markets.

#### Reduced medical costs

Since we have no measure of the men’s out-of-pocket costs for analgesics, medicines, traditional healers, or transportation to health care facilities before the hydrocelectomy, we could not measure the reduction in those expenses as a result of the surgery, thus underestimating the benefits of the procedure.

#### Qualitative effects

We did not quantify the non-monetary cost of pain, mobility restriction, depression, withdrawal from engagement in the community, and reduced quality of life for the patient and his family.[36]

#### Age progression

If the degree of disability imposed by hydrocele were positively correlated with age, then our methodology would underestimate the lifetime earnings loss due to uncorrected hydrocele because we assumed that earnings loss would be the same every year until aging out of the workforce. That assumption may have produced an underestimate of the gains from hydrocelectomy.

All of these omissions reduced the estimate of the lifetime societal cost of filarial hydrocele morbidity and thus understated the benefit of corrective surgery.

### Factors with ambiguous effects on the benefit-cost ratio for hydrocelectomy

#### Seasonal variation in agricultural work

For agricultural workers, the intensity of work typically varies depending on the season. The survey asked about days of work lost in the previous month (November) due to hydrocele. In Malawi, November is at the beginning of the rainy season during which planting must be completed. When work is episodic and the timing of specific tasks is crucial, lost productivity can affect earnings not only for that month, but for the whole year. Seasonal variation in work load is important but the impact of hydrocele on seasonal variations in productivity cannot be definitively estimated.

#### Loss to follow-up and selection bias

Selection bias could have affected responses to follow-up interviews. It was determined that follow-up surveys with 155 men randomly selected from the original cohort of 201 would provide sufficient statistical power to carry out the post-surgery analysis.[36] In the three-month follow-up 152 men were interviewed. At six months, 15 men interviewed in the first follow-up could not be interviewed again because they were travelling, working away from home, or no longer living in the district. That loss to follow-up, especially at the six-month interview could have biased the findings, but the direction of any bias is unknown.

### Conclusion

Our goal was to produce a lower-bound estimate of the benefit-cost ratio for filarial hydrocelectomy. The Discussion addresses 2 factors that might have produced overestimation of the benefit-cost ratio. We argue that those factors were unlikely to be important. We also considered numerous factors grouped into 8 categories that could have produced underestimation of the benefit-cost ratio. We conclude that the effect of the latter could have led to a substantial underestimation of the benefit of surgery.

This analysis of a hydrocelectomy campaign in Malawi shows that the economic benefits of surgery for filarial hydrocele are 24.8 times the cost of the intervention. The benefit-cost ratio is robust to differences in assumptions about the length of working lives and cost of surgery. The cost of hydrocelectomy makes it difficult for poor men in low-income countries to pay for the operation, but the lifetime benefits–to the man, his family, and his community–far exceed the costs of repairing the hydrocele. There is a presumption that hydrocelectomy is too expensive and thus infeasible in low-income regions, but this study demonstrates that the benefits of the procedure far outweigh its costs. Scaling up subsidies to hydrocelectomy campaigns should be a priority for governments and international aid organizations to fulfill the second pillar of the Global Programme to Eliminate Lymphatic Filariasis, preventing and alleviating disability among the many millions of persons already affected.
